# Acquired familial Mediterranean fever associated with a somatic *MEFV* mutation in a patient with *JAK2* associated post-polycythemia myelofibrosis

**DOI:** 10.1186/s13023-015-0298-6

**Published:** 2015-06-30

**Authors:** Yael Shinar, Tali Tohami, Avi Livneh, Ginette Schiby, Abraham Hirshberg, Meital Nagar, Itamar Goldstein, Rinat Cohen, Olga Kukuy, Ora Shubman, Yehonatan Sharabi, Eva Gonzalez-Roca, Juan I. Arostegui, Gideon Rechavi, Ninnette Amariglio, Ophira Salomon

**Affiliations:** Heller Institute of Medical Research, Sheba Medical Center, Tel-Hashomer and Sackler Faculty of Medicine, Tel Aviv University, Tel Aviv, Israel; Hematology Laboratory, Sheba Medical Center, Tel Hashomer, Israel; Department of Pathology, Sheba Medical Center, Tel Hashomer and Sackler Faculty of Medicine, Tel Aviv University, Tel Aviv, Israel; Department of Oral Pathology & Oral Medicine, The Maurice and Gabriela Goldschleger School of Dental Medicine, Tel Aviv University, Tel Aviv, Israel; Cancer Research Center, Sheba Medical Center, Tel Hashomer, and Sackler Faculty of Medicine, Tel Aviv University, Tel Aviv, Israel; Institute of Nephrology and Hypertension, Sheba Medical Center, Tel Hashomer, Israel; Maccabi Healthcare Organization, Petach Tikva, Israel; Internal Medicine D, Sheba Medical Center and Sackler Faculty of Medicine, Tel Aviv, Israel; Department of Immunology, Hospital Clinic-IDIBAPS, Barcelona, Spain; Institute of Thrombosis and Hemostasis, Sheba Medical Center, Tel Hashomer and Sackler Faculty of Medicine, Tel Aviv University, Tel Aviv, Israel

**Keywords:** FMF, *MEFV*, Fever, Somatic mutation, Mosaicism, Autoinflammatory, Myelofibrosis, Polycythemia vera, JAK2

## Abstract

**Background:**

A study was designed to identify the source of fever in a patient with post-polycythemia myelofibrosis, associated with clonal *Janus Kinase 2* (*JAK2*) mutation involving duplication of exon 12. The patient presented with 1–2 day long self-limited periodic episodes of high fever that became more frequent as the hematologic disease progressed.

**Methods:**

After ruling out other causes for recurrent fever, analysis of the pyrin encoding Mediterranean fever gene (*MEFV*) was carried out by Sanger sequencing in peripheral blood DNA samples obtained 4 years apart, in buccal cells, laser dissected kidney tubular cells, and FACS-sorted CD3-positive or depleted mononucleated blood cells. Hematopoeitc cells results were validated by targeted deep sequencing. A Sanger sequence based screen for pathogenic variants of the autoinflammatory genes *NLRP3*, *TNFRSF1A* and *MVK* was also performed.

**Results:**

A rare, c.1955G>A, p.Arg652His *MEFV* gene variant was identified at negligible levels in an early peripheral blood DNA sample, but affected 46 % of the *MEFV* alleles and was restricted to *JAK2*-positive, polymorphonuclear and CD3-depleted mononunuclear DNA samples obtained 4 years later, when the patient experienced fever bouts. The patient was also heterozygous for the germ line, non-pathogenic *NLRP3* gene variant, p.Q705K. Upon the administration of colchicine, the gold standard treatment for familial Mediterranean fever (FMF), the fever attacks subsided.

**Conclusions:**

This is the first report of non-transmitted, acquired FMF, associated with a *JAK2* driven clonal expansion of a somatic *MEFV* exon 10 mutation. The non-pathogenic germ line *NLRP3* p.Q705K mutation possibly played a modifier role on the disease phenotype.

## Background

Polycythemia vera (PV; MIM 263330) is a clonal progressive myeloproliferative disorder primarily characterized by elevation in red blood cells, often with increased myeloid elements. The major pathogenic event in PV is the acquisition of a somatic gain-of-function mutation in the *Janus Kinase 2* gene (*JAK2*; MIM 147796), resulting in erythropoietin independent proliferation of erythroid progenitor cells. Approximately 96 % of PV cases involve the p.V617F mutation in exon 14 of *JAK2* [1], while 3 % involve exon 12, with 37 different mutations described to date [2–5]. Transformation into myelofibrosis and acute leukemia occurs in 10 and <3 % of the patients, respectively, during a 10-year disease course [1].

The Mediterranean fever gene (*MEFV*; MIM 608107) is highly expressed in myeloid cells, particularly in mature granulocytes. This gene codes for pyrin, a cytoplasmatic protein that regulates the maturation and secretion of the proinflammatory cytokines IL-1b and IL-18 in the inflammasome complex [6–9]. Missense mutations in *MEFV* associate with familial Mediterranean fever (FMF; MIM 249100), an autoinflammatory and inherited disorder prevalent in Mediterranean descendants. FMF is characterized by 2–3 days long self-limited attacks of fever, abdominal pain, arthritis and /or pleuritis [10]. The attacks are accompanied by leukocytosis and neutrophil infiltration to synovial membranes. Colchicine is the gold standard treatment for FMF, and is of diagnostic value. Biallelic *MEFV* exon 10 mutations are detected in 50–60 % of FMF patients. In 10–20 % of patients a monoallelic mutation is found, usually manifested with mild symptoms [11–15]. A gain-of-function, leading to an increase in the maturation of proinflammatory cytokines to their secreted forms, was suggested to explain the pathogenicity of these conservative missense *MEFV* mutations [8, 9]. A case of transmission of FMF by bone marrow transplantation from a donor with undiagnosed FMF proved the disease could be acquired through *MEFV* mutated hematopoietic cells [16].

Herein, we present for the first time genetic evidence for naturally acquired FMF, in a middle aged patient who had post-PV myelofibrosis. The patient developed FMF, while a negligible, myeloid restricted somatic *MEFV* exon 10 mutation increased in its mosaicism level. Co-segregation into myeloid cells with a *JAK2* mutation with growth advantage suggests that the *MEFV* mutation was a hitchhiker during clonal expansion.

## Methods

### Patient

The patient is a 59 years old Ashkenazi Jewish female diagnosed with PV at 52 years, and followed at the Sheba Medical Center, Tel Hashomer, IL. Genetic testing for the *JAK2* p.V617F mutation was negative but sequencing of exon 12 revealed the NM_004972.3:c.1608_1640dup variant, known as F537-I546dup+F546L on the protein level [3] and designated p.(Ile546_Phe547insLeuPheHisLysIleArgAsnGluAspLeuIle) according to HGVS nomenclature. This duplication predicts a substitution in the p.Phe547 hotspot residue followed by insertion of ten amino acids in the SH2 linker and pseudokinase JH2 domain. The patient has been treated with phlebotomies when needed and with100 mg acetylsalicylate daily. Four years later the patient’s spleen enlarged dramatically reaching 20 cm and a bone marrow biopsy confirmed transformation into myelofibrosis according to the international working group for myelofibrosis [17]. At that time, the patient developed fever bouts, initially reaching 38 °C and lasting 24 h, at 2 months intervals and a year after occurring at shorter intervals, rising to 39 °C and lasting up to 48 h with occasional abdominal pain and/or muscle aches. The C-reactive protein was elevated during fever, reaching 19 mg/L (normal levels <0.08–5 mg/L). Extensive work up for fever of unknown origin failed to detect infection, malignancy, collagen disease or thrombotic events. Clinical diagnosis of FMF was then considered. Surprisingly, a rare mutation in the *MEFV* gene was detected and daily treatments with 1 mg colchicine abated the fever. A recent development of mild renal failure with moderate proteinuria led us to perform a kidney biopsy.

### Samples

Polymorphonuclear (PMC) and mononuclear cells (PBMC) were isolated by density gradient centrifugation using Histopaque 1077 (Sigma-Aldrich, St. Louis, MO), yielding a pellet of PMC and a PBMC cell band at the Histopaque/plasma interphase. Genomic DNA was prepared from whole and isolated fractions of peripheral blood cells (PBC), and from buccal cells using a commercially available kit (iNtRON biotechnology, Kyungki-Do, Korea). Genomic DNA from kidney tubule cells was purified with a column-based method (QIAamp® DNA Micro Kit; Qiagen). This study was approved by the ethics committee at the Sheba Medical Center, and was performed according to the declaration of Helsinki.

### Genetic analyses

The JAK2 p.V617F mutation was excluded [18]. *JAK2* exon 12 was PCR-amplified with flanking primers. Two distinct gel bands on a 2 % agarose gel were excised, purified by MinElute Gel extraction kit (QIAGEN, Hilden, Germany) and sequenced. Amplicons *of MEFV* exons 1–10 (LRG_190t1), *NLRP*3 exon 3 (LRG_197t1), *TNFRSF1A* exons 2–4 (LRG_193t1), and *MVK* exons 2–11 (LRG_156t1) were sequenced after Exo-Sap treatment. Sequencing reactions were performed using the Big Dye Terminator kit (Applied Biosystems, California, USA) on an ABI 3130XL automated sequencer, and the sequences were analyzed with BioEdit or blast software.

For targeted deep sequencing, a first PCR reaction surrounding the c.1955 nucleotide change of *MEFV* was performed in triplicate. Barcodes were incorporated to amplicons during a second, nested round of amplification. Amplicons were then purified using Agentcourt AMPureXP beads (Beckman coulter, Nyon, Switzerland) and the DNA quantified using High Sensitivity DNA kits for Bioanalyzer (Agilent technologies, Ca, USA). Sequencing and analyses were performed using the 400 bp kit on a PGM system, Ion Torrent server and Ion Reporter software, according to manufacturer’s instructions (Ion Torrent, Life Technologies, Guilford, Connecticut, USA). Coverage at the c.1955 position was at least ×3000.

### Restriction Site Length Polymorphism (RFLP) analysis

Exon 10 RFLP of the c.1955G>A *MEFV* variant was performed with Fnu4hI enzyme (New England Biolabs, Ipswich, MA, USA) following manufacturer’s instructions.

### FACS cell sorting

PBMC were immunostained with PE-conjugated anti-CD3 (UCHT1) mAbs from BD Biosciences (San Jose, CA) for 15 min at room temperature. Two cell populations, CD3+ T cells and CD3-depleted PBMC were sorted with high purity (>95 %) with a stringent multiparametric discrimination algorithm, by FACSAria digital cell sorter (BD Biosciences).

### Pathology

Bone marrow biopsies obtained in 2009 and 2012 showed morphological features of myelofibrosis with increased reticulin fibers (grade 2). Kidney biopsy showed diffuse mesangioproliferative and focal endocapillary proliferative glomerulonephritis. Immunofluorescence showed IgM and C3+ granular stain without clonality. Congo red staining was negative for amyloid.

### Laser capture microdissection

Five-micrometres thick paraffin embedded kidney biopsy sections were placed on membrane-coated slides (PALM, Munich, Germany), heated at 60 °C overnight and stained with hematoxillin and eosine. Tubular cells were dissected and catapulted onto a microfuge tube lid on a robotstage microscope equipped with a 337-nm pulsed laser microbeam (PALM, Munich, Germany) with a single laser shot.

## Results

**Fig. 1:**
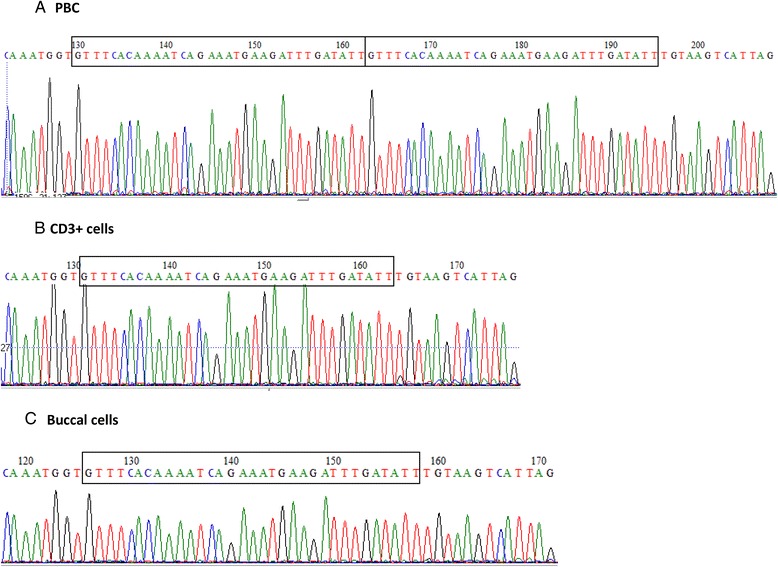
Sanger Sequencing results of *JAK2* exon 12 DNA sequence (NM_004972.3). DNA sequencing **a** of PBC presenting the c.1608_1640dup nutation **b**-**c** of CD3+ cells and buccal cells presenting wild type DNA sequence of the gene. The *rectangle* contains the exon 12 sequence which is duplicated in the PBC sample and not in the CD3+ T cells or the buccal cells

**Fig. 2:**
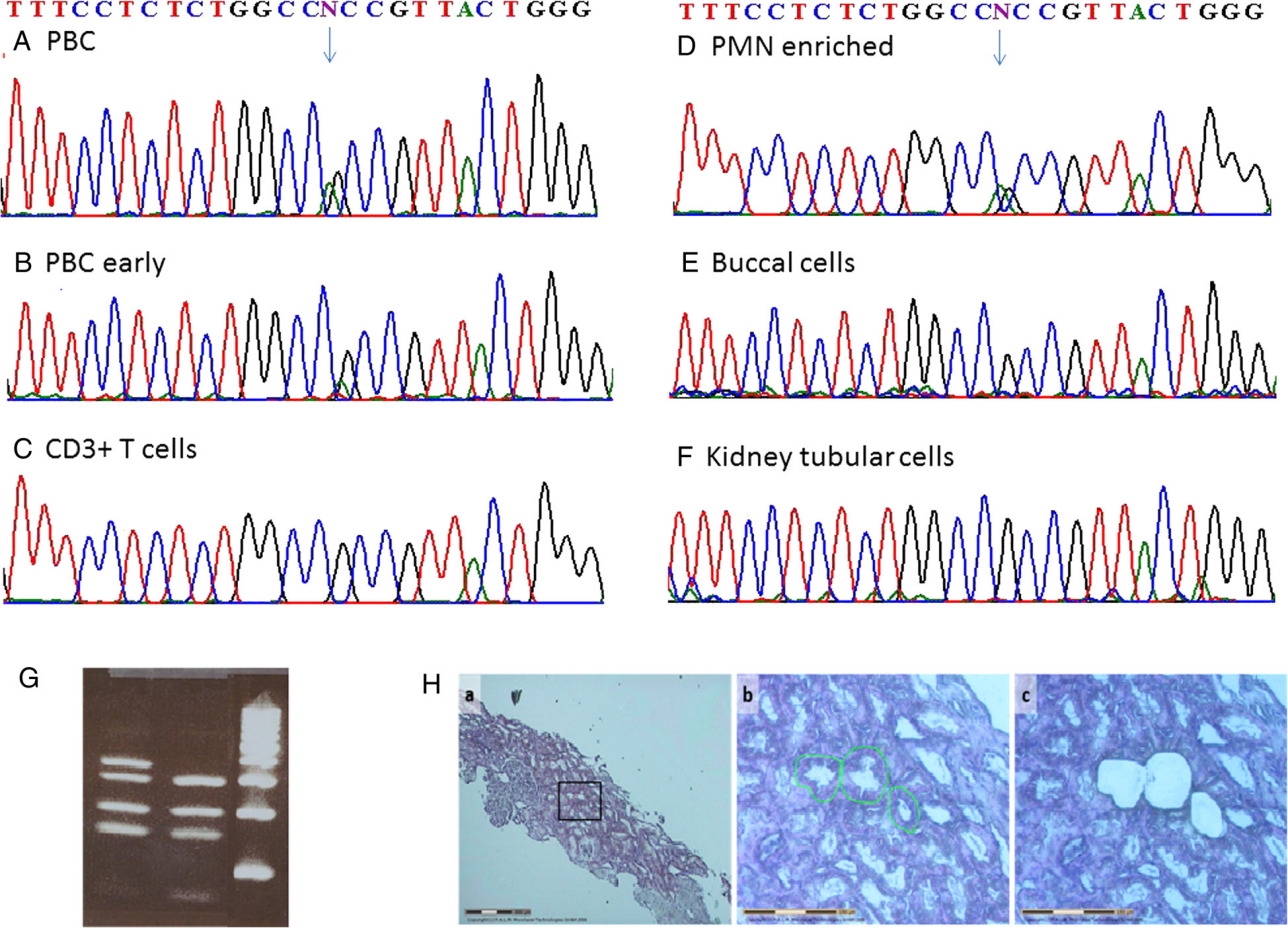
Detection, expansion and somatic distribution of the *MEFV* c.1955G>A mutation demonstrated by Sanger sequencing. **a** PBC DNA sample of the patient from 2009 **b** an earlier sample from 2005 **c** CD3-positive T cells, **d** PMC enriched fraction, **e** buccal cells, **f** kidney tubule cells. **g** Fnu4h1 RFLP of *MEFV* exon 10 visualized on a 4 % agarose E-gel. The *MEFV* mutation abolishes the second of three restriction sites yielding a longer, 350 base pairs fragment. **h** Small power photomicrograph demonstrating a glomerulus and tubular cells. **h**-*a* High power photomicrograph demonstrating a desiccated tubule cell, **h**-*b* before and **h**-*c* after laser capture microdissection procedure (hematoxylin and eosin stain magnification ×400)

The patient presented with post-PV myelofibrosis involving a rare duplication in exon 12 of *JAK2* (Fig. 1a) previously described in one patient [3]. The occurrence of recurrent fever in the absence of any known underlying cause led us to search for *MEFV* gene mutations. Her Ashkenazi origin, middle aged onset and lack of family history of FMF were not conflicting, as these have been previously noted [19, 20]. The NM_000243.2:c.1955G>A mutation (rs28940581) was found in exon 10 (Fig. 2a and 2g) predicting a p.Arg652His substitution in the PRYSPRY domain of pyrin. In the domain 3D model, the R652 residue localizes to the β5 strand end within sheet B. This variant was recently reported in a patient affected by Crohn’s disease [21], a well-established comorbidity with FMF [22]. A somatic origin for the p.Arg652His mutation was suggested to explain the post-PV MF associated recurrent fever. We therefore tested a DNA sample preceding the onset of fever by 4 years, when a search for a *JAK2* mutation was performed. In the early sample the mutated *MEFV* variant was repeatedly observed at a negligible level by Sanger sequencing (Fig. 2b) whereas the *JAK2* exon 12 extra gel band was highly visible. To further characterize the somatic origin of the *MEFV* mutation, we performed cell sorting on PBMC. Both the *MEFV* p.Arg652His and *JAK2* exon 12 mutations were present in CD3-depleted peripheral blood mononuclear cells and the polymorphonuclear cells, but not in CD3+ T lymphocytes (Figs. 1b, 2c and d). Of note, *JAK2* mutations are reported undetectable in T cells. Targeted deep sequencing confirmed these results showing a mutated *MEFV* allele frequency of 46 % in peripheral blood and in polymorphonuclear cells, 27 % in CD3-depleted PBMC and 0 % in CD3+ T cells. In the DNA of buccal cells neither the *JAK2* nor the *MEFV* mutations were detected (Figs. 1c and 2e).

After the control of fever with colchicine treatment for almost a year, the patient developed heavy proteinuria with mild renal failure requiring kidney biopsy. We expected to detect glomerulosclerosis, a known late complication of myeloproliferative disorder (MPD) or secondary amyloid nephropathy related to FMF. Unexpectedly, the kidney biopsy showed membranoproliferative glomerulonephritis and Congo red staining was negative for amyloid. The somatic origin and restriction of the *MEFV* mutation to hematopoietic cells was then re-challenged and reaffirmed on single tubule cells derived from kidney biopsy (Fig. 2f and h). These results support the somatic nature of both *JAK2* and *MEFV* mutations, their restriction to a myeloid clone and an increase in the somatic *MEFV* mutation burden associated with myelofibrosis progression.

Finally, sequencing of three other known autoinflammatory genes revealed one heterozygous, non-pathogenic variant in the *NLRP3* gene, NM_004895.4:c.2113C>A, p.Gln705Lys (historically called Q703K). This variant was detected in both the early and late peripheral blood DNA samples as well as in CD3+ T cells, and was concluded to be a germ line mutation.

## Discussion

The genetic analysis of a post-PV myelofibrotic patient clinically diagnosed with FMF [10] revealed a unique somatic *MEFV* variant which, in parallel to the development and aggravation of FMF attacks, underwent expansion. In addition, the patient had a known, prevalent germ line *NLRP3* variant, unrelated to a specific autoinflammatory disease, yet reported to lead to an overactive *NLRP3* inflammasome, or suggested to be a low penetrance variant, which hitherto was clinically silent [23, 24].

Several findings support a pathogenic role for the rare somatic *MEFV* mutation. First, the p.Arg652His variant was the sole variation in the coding region of the gene. Second, the somatic variant was confined to the disease effector cells, and third, the level of *MEFV* mosaicism increased in association to the development and progressive aggravation of the FMF attacks.

On the other hand, we argue against a pathogenic role for the germ line p.Q705K *NLRP3* variant in the development of FMF, since the patient did not have an inflammatory phenotype prior to the expansion of the *MEFV* mutation, nor is this variant known to cause FMF. At most, it may have up-regulated the proinflammatory effect of the *MEFV* mutation, serving as a modifier gene in the myeloid cells [25].

Conceivably, the acquisition of a pathogenic mutation in the *MEFV* gene in somatic cells is more frequent than heretofore observed. It remains unnoticed however, when present in only a few cells. Somatic expansion of the *MEFV* mutation made the p.Arg652His mutation clinically visible in our patient, and tight co-segregation supports a *JAK2* driven expansion of an *MEF*V mutated myeloid clone, irrespective of the order in which the two mutations occurred.

It is possible that the magnitude of inflammation was up-regulated in our patient by a combined effect of the rare exon 12 *JAK2* mutation and the germ line, p.Q705K modifier variant, on the inflammasome priming via the ERK tract [2, 25]. It may also be that in other instances recurrent FMF fever bouts have been misclassified as constitutional symptoms, related to the primary disease.

Myeloid restricted, non-malignant somatic mosaicism with low and variable degree (8–27 %) has recently been reported in the *NLRP3* gene of patients with the autoinflammatory, variant type Schnitzler syndrome, an urticarial and systemic inflammation disease with monoclonal gammopathy [26], and in a case of cyropyrin associated periodic syndrome (CAPS) [27]. *NLRP3* mosaicism affecting several tissue types is more frequently found, especially in NOMID/CINCA patients who test negative for a heterozygous germ line mutation [28, 29]. Lastly, a case of somatic mosaicism has been reported in a patient with Blau syndrome [30]. To the best of our knowledge the present case is the first demonstration of a somatic *MEFV* mutation, expanding the spectrum of autoinflammaory diseases caused by somatic mosacism.

In summary, this paper highlights a diversion of nature in the course of post-PV myelofibrosis. For such a diversion to occur two concomitant events are necessary: A proliferation driver mutation should evolve in myeloid cells and a passenger pathogenic mutation should occur in a gene which is normally expressed in the proliferating clone. Whether the driver and the hitchhiker mutations converged in our case to regulate a common pathogenic pathway awaits further proof. The phenomenon described herein may also elucidate the pathogenesis of recurrent fever of unknown origin frequently detected in other malignancies.

## Conclusions

This work provides clinical and genetic evidence for a unique pathogenic route that leads to acquired FMF. A somatic, myeloid restricted mutation in the Mediterranean fever gene of a patient with post-PV myelofibrosis expanded from negligible to 46 % of total *MEFV* alleles in peripheral blood cells, parallel to the development of colchicine responsive inflammatory fever bouts. Co-segregation of the *MEFV* and *JAK2* exon 12 mutations into myeloid cells suggests the *MEFV* mutation was a passenger in a *JAK2* driven proliferating clone. Other autoinflammatory diseases may be acquired due to somatic mutations in additional clonal myeloproliferative diseases.
